# A new strain of *Neowestiellopsis* (Hapalosiphonaceae): first observation of toxic soil cyanobacteria from agricultural fields in Iran

**DOI:** 10.1186/s12866-022-02525-x

**Published:** 2022-04-18

**Authors:** Bahareh Nowruzi, Lenka Hutárová, Itzel Becerra Absalón, Liwei Liu

**Affiliations:** 1 Department of Biotechnology, Science and Research Branch, Islamic Azad University, Tehran, Iran; 2 Department of Biology, Faculty of Natural Sciences, University of Ss. Cyril and Methodius in Trnava, Trnava, Slovakia; 3 Laboratorio de Ficología, Facultad de Ciencias, Universidad Nacional Autónoma de México, Ciudad de México, Mexico; 4 Department of Marine Pharmacy, College of Food and Pharmaceutical Sciences, Ningbo University, Ningbo, 315211 P. R. China; 5 Stake Key Laboratory of Natural and Biomimetic Drugs, Peking University, Beijing, 100191 P. R. China

**Keywords:** Cyanobacteria, Internal transcribed spacer (ITS), *Mcy* genes, *Neowestiellopsis*, Phylogenetic analysis, 16S rRNA gene

## Abstract

**Background:**

In the present research, challenges arose when many reports have been published on the poisoning of humans due to the ingestion of crops of *Crataegus* plants contaminated with cyanobacterial toxins. The discovery of several poisonings around agricultural zones prompted us to study the toxic compounds in a strain of *Neowestiellopsis* which is the most abundant in the agricultural zones of Kermanshah province of Iran, using a polyphasic approach. Molecular procedure was followed to study these strains deeply.

**Material and methods:**

To elucidate their systematic position, besides the 16S rRNA gene, the analyses of molecular toxicity markers, namely *nos, mcy G, mcy D* and internal transcribed spacer (ITS), were also used.

**Results:**

Based on the results, for the first time, we record the presence of a gene cluster coding for the biosynthesis of a bioactive compound (Nostopeptolides) that is very rare in this family and the presence of toxic compounds (microcystin), which might account for the poisoning of humans.

**Conclusions:**

This case is the first observation of a toxic soil strain from the genus *Neowestiellopsis* from agricultural fields in Iran.

**Supplementary Information:**

The online version contains supplementary material available at 10.1186/s12866-022-02525-x.

## Background

Cyanobacteria are a very varied group of prokaryotes. They perform oxygenic photosynthesis, like that detected in higher plants and algae [1]. They colonized a wide range of biotopes and contain different biologically active compounds, which could improve plant growth and are considered to improve the physicochemical characteristics of soils [2, 3]. However, the use of surface water polluted with cyanotoxins for crop irrigation or dried toxic cyanobacteria cells as fertiliser may be a source of toxin pollution in soil [4, 5]. Bittencourt-Oliveira et al. [6] showed that irrigation of crop plants by water polluted with microcystins is not only a commercial problem but also a public health issue because of the risk of food pollution. This way of contact needs careful checking by the responsible experts [6]. The toxins present in the soil solution are also accessible for uptake by soil organisms, such as plants. For instance, Pflugmacher et al. [7] established a fast uptake of microcystin by an aquatic plant *Phragmites australis*. Furthermore, surface waters used for agricultural practices typically comprise dense toxic blooms of cyanobacteria [8]. Only 14 out of the 40 cyanobacterial genera have been identified as toxic, even though 50–75% of cyanobacterial blooms are toxic. recognised. Cytotoxins (e.g., mirabilene, scytophycins, toyocamycins, isonitriles, indocarbazoles, tubercidin, paracyclophanes, acutiphycins, tolytoxins and tentazolesand) are not dangerous to organs in the human body, whereas biotoxins damage organ systems [9]. Cytotoxins are important for their antitumor and antimicrobial ability and may hypothetically be used for therapeutic purposes [10, 11]. Once in the soil, cyanotoxins may be moved again to water bodies by runoff, leaching, and drainage processes, or can be gathered in the soil. Consequently, they could be absorbed by plants straight from the soil, or they can produce surface pollution which might cause contamination. Though, it appears that the adsorption of these toxins is usually low, which increases their bioavailability for plants. As well as potential effects on human health, higher levels of cyanotoxins in soils can negatively affect animal health, plant vigour, microbial processes, and general soil condition [8]. Long-term contact with some quantities of cyanotoxins may be causally related to human diseases. Besidesthe effect on humans and animal health, cyanotoxins can also exert harmful impact on other biota including plants and invertebrates, including oxidative stress, inhibition of photosynthesis, leaf necrosis, and growth [12].

In cyanobacteria, compounds such as thrombin inhibitor spumigin E, nostopeptolide, microcystin inhibitor nostocyclopeptide M1 and protein kinase inhibitor brisebromoamide were identified [13]. For instance, the nostocyclopeptide’s cyclic peptide may inhibit the hepatotoxic action of nodularin and microcystin [14]. The nostopeptidolite was first described in the terrestrial strain *Nostoc* sp. CSV 224, where it may show an important role in cellular differentiation [15]. The synthesis of nostopeptolide has been proven by Luesch et al. [16] at enzymatic and genetic levels, and the coding genes for the enzymes were named *nos*. We hypothesize that the screening of Hapalosiphonaceae for the presence of the biosynthetic genes *nos*, could lead to the identification of nostopeptolide, which is very rare in this family.

The increase of human poisonings in the agricultural zone of Kermanshah province of Iran via feeding on crops of plants from the genus *Crataegus* has led to an increased research interest and public awareness of harmful toxic cyanobacteria. Kermanshah has a moderate and mountainous climate, with rainy winters and moderately warm summers. On the other hand, agricultural zones in Kermanshah province represent a very extensive area with sub-tropical climate characterized by a long, dry summer, with an air temperature often exceeding 40 °C during July and August. Rainfalls (only 200 mm) are restricted to autumn, winter, and spring. Despite the semi-desert climate, the relative humidity is relatively high. These favourable conditions are suitable for the growth of cyanobacterial strains. The knowledge of the diversity of toxic cyanobacterial strains in the agricultural zone of Iran, where human poisoning occurs, is still incomplete.

Genus *Neowestiellopsis*, originally described by Kabirnataj et al. [17] from Mazandaran (Iran), belongs to order Nostocales and family Hapalosiphonaceae [18]. Strains of this genus can be found in both paddy fields and agricultural zones, and, due to their ability to fix nitrogen, some strains have an important role in agriculture [19, 20]. In Iran, polyphasic studies of cyanobacteria are still scarce [21–26] and limited to phylogenetic studies of genes encoding proteins involved in the biosynthesis of bioactive compounds in paddy fields and freshwater regions (for e.g., [17–31]). Therefore, the aim of the present study was to characterize the strain at the phenotypic and molecular levels and identify the toxic gene cluster of bioactive compounds present in one potentially toxic strain of the genus *Neowestiellopsis* for the first time isolated from agricultural zones of Iran. This polyphasic study led to the identification of a potentially toxic strain from the genus *Neowestiellopsis*, which contributes to the increase of knowledge on cyanobacterial diversity from this region.

The goal of the present study was determining the taxonomic position of a new cyanobacterial strain of *Neowestiellopsis* isolated from the Kermanshah province soil samples combine the methods of morphological study and multiple genes sequence analyses, as well as deciding if the strain could be toxic.

## Results

### Phylogenetic analyses

**Fig. 1:**
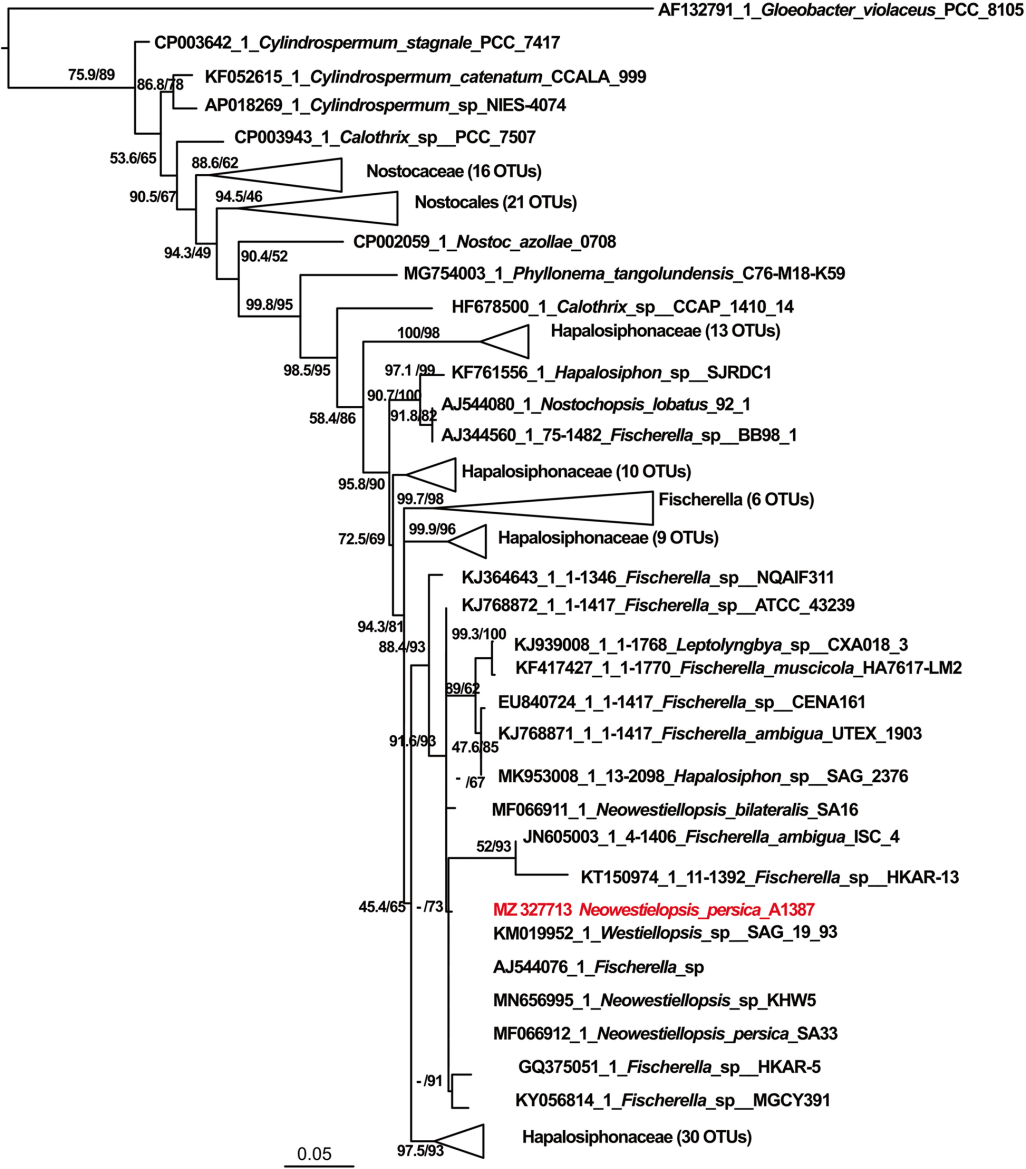
Phylogenetic relationships between studied strain (Highlighted in red) and related cyanobacteria based on 16S rDNA sequences with *Gloeobacter violaceus* PCC 8105 (AF132791) as out group. Numbers near nodes indicate standard bootstrap support (%)/ultrafast bootstrap support (%) for ML analyses

To establish the taxonomic position of our strain, ann analysis of a 16S rRNA gene fragment with 2102 bp was performed. Comparative analyses of the obtained sequences revealed a 100% homology with *Neowestiellopsis persica* SA33 (MF066912.1), with the difference only in one nucleotide. The clade, in which our strains belong, is composed by other *Neowestiellopsis* strains (*Neowestiellopsis* sp. KHW5 (MN656995) and *Neowestiellopsis persica* SA33 (MF066912) and *Fischerella* strains (*Fischerella ambigua* ISC 4 (JN605003); *Fischerella* sp. HKAR-13 (KT150974); *Fischerella* sp. (AJ544076); *Fischerella* sp. HKAR-5 (GQ375051) and *Fischerella* sp. MGCY391 (KY056814) and *Westiellopsis* sp. SAG 19 93 (KM019952) (Fig. 1), however they form a separate branch.

In the phylogeny based on *mcy D* (Fig. S3) the same pattern was observed, the strains are placed in the same cluster, being the *Fischerella* sp. NQAIF311 (KJ364644) and *Fischerella* sp. CENA161 (JQ771634). However, when we investigated the phylogeny based on *mcy G* (Fig. S2), the studied strain makes a close clade with *Hapalosiphon hibernicus* BZ-3-1 (EU151893). Moreover, phylogenetic tree based on *nos* (Fig. S4) genes, while the studied strain makes a big cluster together with *Nostoc* sp. _*Peltigera membranacea* cyanobiont N6 (CP026681), *Nostoc* sp. *Peltigera membranacea* cyanobiont (GU591312), *Anabaena* sp. YBS01 (CP034058), *Trichormus variabilis* 0441 (CP047242) and *Anabaena variabilis* ATCC 29413 (CP000117), it falls into separate clades. However, when we investigated the phylogeny based on *mcy G* (Fig. S2), the studied strain makes a close clade with *Hapalosiphon hibernicus* BZ-3-1 (EU151893). Moreover, phylogeny tree based on *nos* genes (Fig. S4), there are not yet reliable sequences of the family with which to compare our sequences, therefore the studied strain falls into separate clades, while the studied strain makes a cluster together with *Nostoc* sp. *Peltigera membranacea* cyanobiont N6 (CP026681), *Nostoc* sp. *Peltigera membranacea* cyanobiont (GU591312), *Anabaena* sp. YBS01 (CP034058), *Trichormus* variabilis 0441 (CP047242) and *Anabaena variabilis* ATCC 29413 (CP000117).

### Phenotypic characterization

#### Morphological characterization

**Fig. 2:**
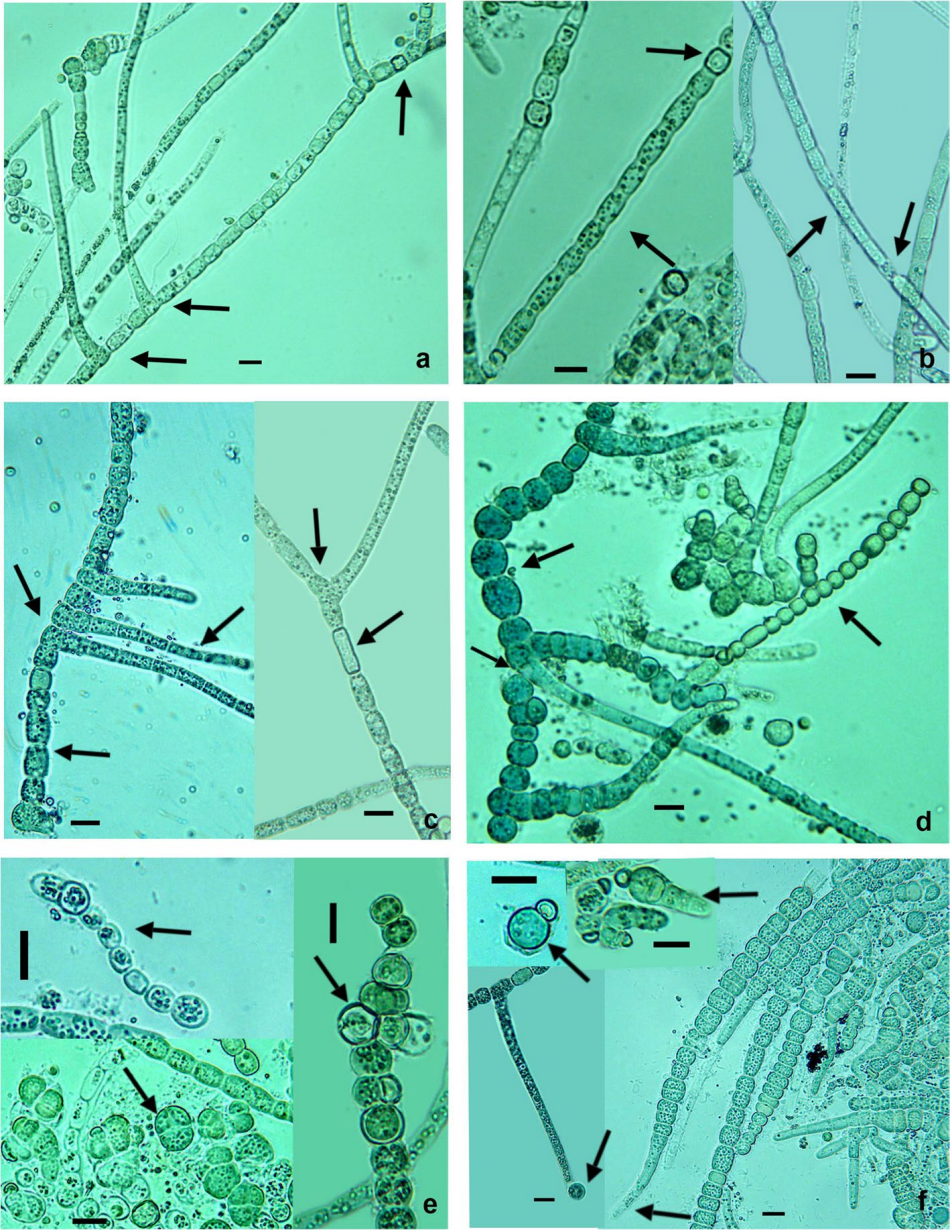
Microphotographs of the new *Neowestiellopsis* isolated from Iran

Microscopical observation of the materials allowed the identification of several cyanobacterial filaments, with long true type T branches, separated for two vegetative cells (down arrows) and intercalary, isodiametric heterocytes (up arrow), typical for the Hapalosiphonaceae family (Fig. 2a). In the filaments, the heterotrichy was observed, with differences in the cell shapes from main filament and branches (Fig. 2a, c, d). Sheaths were observed initially fine, colorless (Fig. 2a), later thicker, firm, or gelatinous, black colored (Fig. 2b), sometimes open at the ends.

With increasing age of filaments there are significant increases in the number of main and branching filaments terminating in an empty sheath (Fig. 2a, b). Usually erect, unilateral T-type branches arise from the main filament (Fig. 2a, c, d), they are originated sometimes from two near cells. The two near cells are connected together, (Fig. 2c) or they are separated by two or more cells (Fig. 2a, c, d), or heterocytes. Also, V-type (Fig. 2c) and simple false (Fig. 2d) branches can be distinguished, but they are rare (Fig. 2d). The main trichomes were formed for spherical or cylindrical cells (0.7–1.1 longer than wide), 5.0–27.5 μm Length × 6.3–15.5 μm Width, with constricted cross walls (Fig. 2a, c, d). The branches usually had cylindrical cells (3–8 longer than wide), 3.75 μm length × 4.5–6.0 μm width, with not constricted at the crosswalls (Fig. 2a, c, d). The apical cells were observed rounded at the ends (Fig. 2a, c, d). Cell contents were slightly granular and sometimes vacuolated (Fig. 2a, b, c). Sometimes neritic cells could be found in the main filaments (Fig. 2b). Heterocysts were intercalary and terminal. Intercalary heterocysts were subspherical (Fig. 2a, b) and cylindrical (Fig. 2c) or even compressed (shorter than broad) in main filaments, 6.25–16.25 μm length × 6.5–11.5 μm width, in branches 2.5–6.25 μm length × 4.8–8.5 μm width. Studied strain may eventually differentiate series of spherical, thick-walled cells that are akinetes (Fig. 2d), 6.25 μm length × 3.75-5 μm width. Reproduction occurs via hormogonia (up left arrow in Fig. 2d), down left arrow akinets germination and right arrow multicellular filament formed by akinets germination (Fig. 2e), akinetes (Fig. 2d, e) and down left arrow at (Fig. 2f) shows monocyte formation, up left arrow monocyte with heterocyte, up right arrow (at Fig. 2f) shows little heteropolar filaments with basal heterocyte, in the right it shows heteropolar filaments with attenuated ends (downright arrow, Fig. 2f).

Typically, hormogonia are morphologically distinct from the main branches, 5.0 – 8.75 μm width and 15 – 72 cells in each hormogonium. The monocyte was a spherical cell, 3.5 – 5. 5 μm of diameter.

When we compared the morphology of studied strain with *Neowestiellopsis persica* SA33 (MF066912.1) and *N*. *bilateralis* SA16, we found that lots of difference in morphological characterization (Table 1). The branching of *Neowestiellopsis bilateralis* was found on both sides of main axis, however there was on only one side on our studied strain, more like *N. persica*. Our strain presented V and T type branching while *N. persica* and *N. bilateralis* only had T type branching. In *N. persica* SA33 was seen biseriate development, terminal cells of branches were tapered toward apex and first cell of branch adjacent to main filament was irregular shape, however these characteristics were never seen in our studied strain.

**Table 1:** Morphological observation of studied strain. The latter was based on previously published photomicrographs

		***Neowestiellopsis*** A1387	***N. persica*** SA33	***N. bilateralis*** SA16
**Thallus**		Creeping and erect filaments	The main filaments were thicker and creeping than the branches	The main filaments were thicker and creeping than the branches
**Heterotrichy/main axis/branches**		+/U & B/U	*+/*U & B/ U & B	+/U/U
**Color of Thallus**		olive green	greenish	bluish green
**Branching**		T-type only one side of main axis and V-type.	T-type only one side of main axis.	T-type both sides of main axis.
**Vegetative cells in main filaments**		spherical to rectangular, 0.7–1.1 × longer than wide, 6.5–13.5 μm length, 6.3–15.5 μm width	width usually much greater than length, 4.39-5.41 μm length, 7.52-9.29 μm width	square, cylindrical or barrel shape, 3.64-7.36 μm length, 4.8-10.62 μm width
**Vegetative cells in branching filaments**		spherical or slightly oblong, 3–8 × longer than wide, 11.2–29.5 μm length, 4.5–6.0 μm width	Irregular-shaped cells with some being squeezed from both sides, 6.13–6.19 μm length, 6.66–6.73 μm width	Irregular shaped cells with some being squeezed from both sides, 5.92–5.99 μm length, 6.33–6.44 μm width
**Heterocytes In main filaments**		elongate, spherical, or even compressed (shorter than broad) intercalary 10.0–22.5 μm length × 6.5–11.5 μm width	Irregular shaped; Large cells and curved on the width,7.82–7.88 μm length, 10.82–10.89 μm width	Irregular shaped; Large cells and curved on the width, 8.00–8.09 μm length, 10.24–10.41 μm width
**Heterocytes In branching filaments**	**Tr**	7.3–8.0 μm length × 4.8–8.5 μm width	–	–
	**I**	5.3–6.0 μm length × 2.8–3.5 μm width	Large sized; always smaller than those of the main branches, 3.32–3.47 μm length, 4.30–4.38 μm width	Large sized; always smaller than those of the main branches, 6.92–6.95 μm length, 8.03–8.08 μm width
**Akinetes**		Oblong, mainly in chains, 5.0–6.0 μm broad, 6.5–11.0 μm Length.	not observed	Not observed
**Branching**		T and V	T	T
**Multiplication**		HG, A, Monocite	HG	HG,

^a^Type of thallus branching *T* T-branching and *V* V-branching)

^b^*HG* Hormogonia

^c^*A* Akinetes

^d^Heterotrichy that indicates differences in the shape of the cells of the main and secondary branches [*+* Clear differences, *U* Uniseriate, *B* Biseriate]

^e^heterocyst position (*Tr* Terminal, *I* Intercalary)

Also, in both *Neowestiellopsis* species, the main filament cells giving rise to branches had irregular-shaped cells with some being squeezed from both sides, but there were not any irregular-shaped cells in studied strain and in total the mean size of vegetative cells, of both *N. bilateralis* and *N. persica*, were smaller than the studied strain, however the size of heterocytes in main filaments and in branched in both strains were in nearly the same range.

In our strain, the akinetes and monocyte reproductive cells were observed, but these were not reported for the other species of *Neowestiellopsis.*

#### Life cycle description

In this strain was observed three reproduction types, from hormogonia, monocytes and akinetes (Fig. S1). The hormogonia were thinner than the main and branches filaments. They were formed in the apical regions of branches and are released by gliding through an opening at the end of sheaths. The hormogonium grew dividing generally in a single plane until forming a typical vegetative filament (Fig. S1a, b, j). The akinetes were formed in the apices of filaments, then they were released and each akinetes germinated by three planes of division, which formed a multiseriate filament. Later, at one of the ends of this filament, it began to divide into a single plane, so mature filaments with a multiseriate end and the rest of the uniseriate filament could be observed (Fig. S1c, d, e, f). The last reproduction type was rare; the monocyte was formed in the apex of branches. In the first division of the monocyte (germination) formed one terminal heterocyte and then the next end the cell divided until to form a little heteropolar filament 2 to 5 cells (like to heteropolar hormogonium). This little filament grew to form a heteropolar filament to 20 cells approximately, apparently this heteropolar filament after will also form typical vegetative filaments (Fig. S1f, g, h, I, j).

#### 16S-23S rRNA ITS secondary structure

**Fig. 3:**
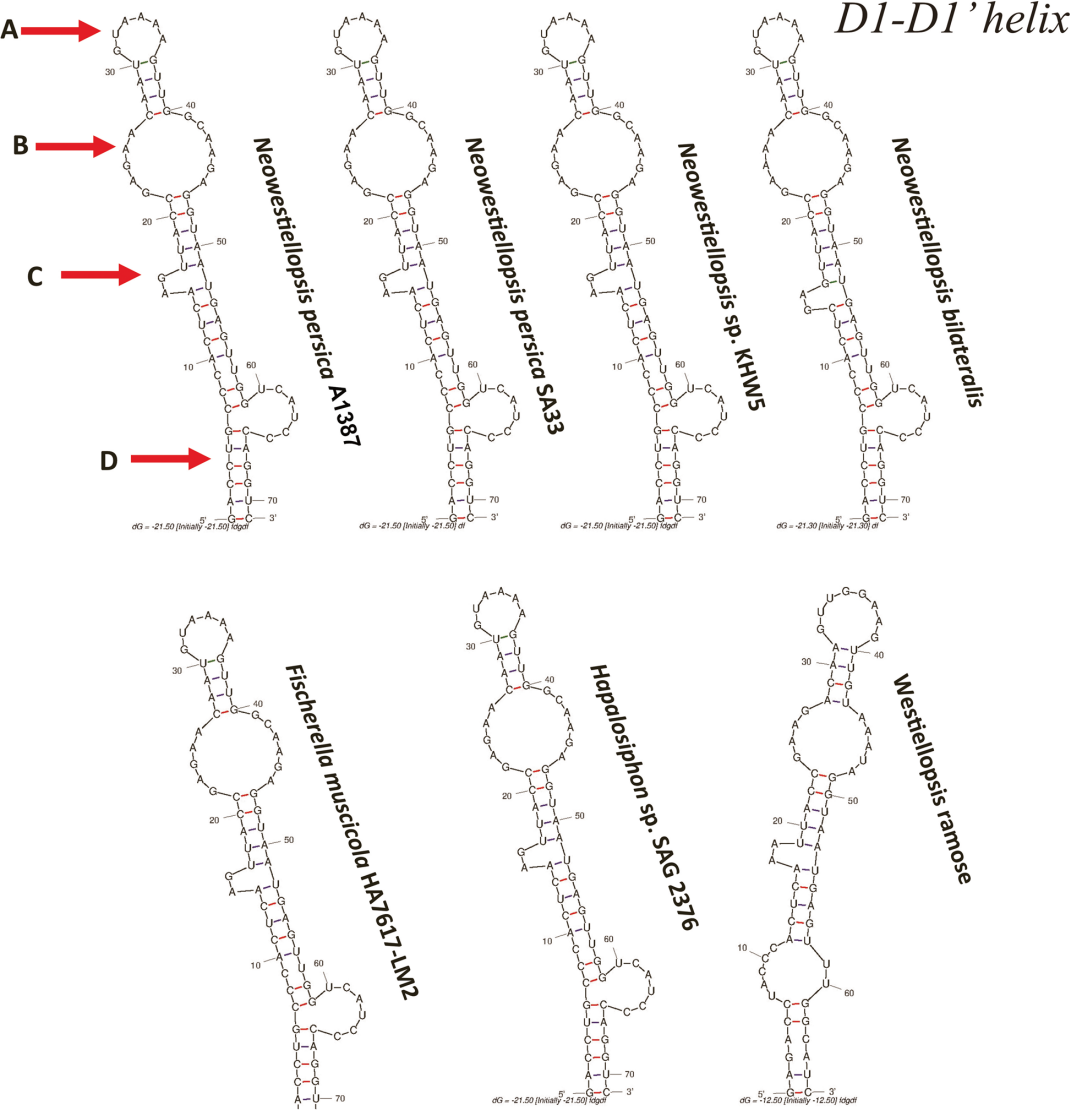
Predicted secondary structures for the D1-D1’ helices of studied strain and related taxa. Secondary structures generated from M-fold web server (version 2.3), temperature: 37 °C default; structure: untangled loop fix. Terminal Bilateral Bulge (A), Bilateral Bulge (B), Unilateral Bulge (C), and Basal Clamp (D)

Six reference sequences were used to search for ITS secondary structure. According to Johansen et al. [32], nine different areas (D1-D1’ helix, D2, D3, tRNA^Ile^ and tRNA^Ala^, BOX B, BOX A, D4 and V3) were found in the ITS secondary structure of studied strains of cyanobacteria and they are important for phylogenetic purposes. Unfortunately, the ITS regions of *Neowestiellopsis persica* SA33 (MF066912.1), *Neowestiellopsis bilateralis* SA16 (MF066911.1) and *Westiellopsis ramose* HPS (KY883375.1) were not sequenced completely and there was only the D1-D1’ helices for comparison. For this reason, we have only compared the ITS regions of the studied strain with *Neowestiellopsis* sp. KHW5 (MN656995.1), *Fischerella muscicola* HA7617-LM2 (KF417427.1) and *Hapalosiphon* sp. SAG 2376 (MK953008.1) (Tables S3, S4 and S5). The D1-D1’, Box-B and V3 ITS regions of all studied strains are revealed to be very different in terms of length and shape (Fig. 3; Tables S3, S4 and S5). The D1-D1’ region of was include a terminal bilateral bulge (A), bilateral bulge (B), unilateral bulge (C), and basal clamp (D) (Fig. 3). The lengths of D1-D1’ helix varied from 61 nt (*Neowestiellopsis persica* SA33) to 71 nt (*Neowestiellopsis bilateralis* SA16, *Neowestiellopsis* sp. KHW5, *Fischerella muscicola* HA7617-LM2 and *Hapalosiphon* sp. SAG 2376) (Table S3), with studied strain showing a helix length of 71 nt (Fig. S3; Table S3). The basal stem revealed to be the same for all studied strains (5′- GUCCAG−− CAGGUC–- 3′), except for *Neowestiellopsis bilateralis* SA16, which showed a different basal stem (5′–- CCAGAG−− GGCAUC–- 3′) (Fig. 3).

**Fig. 4:**
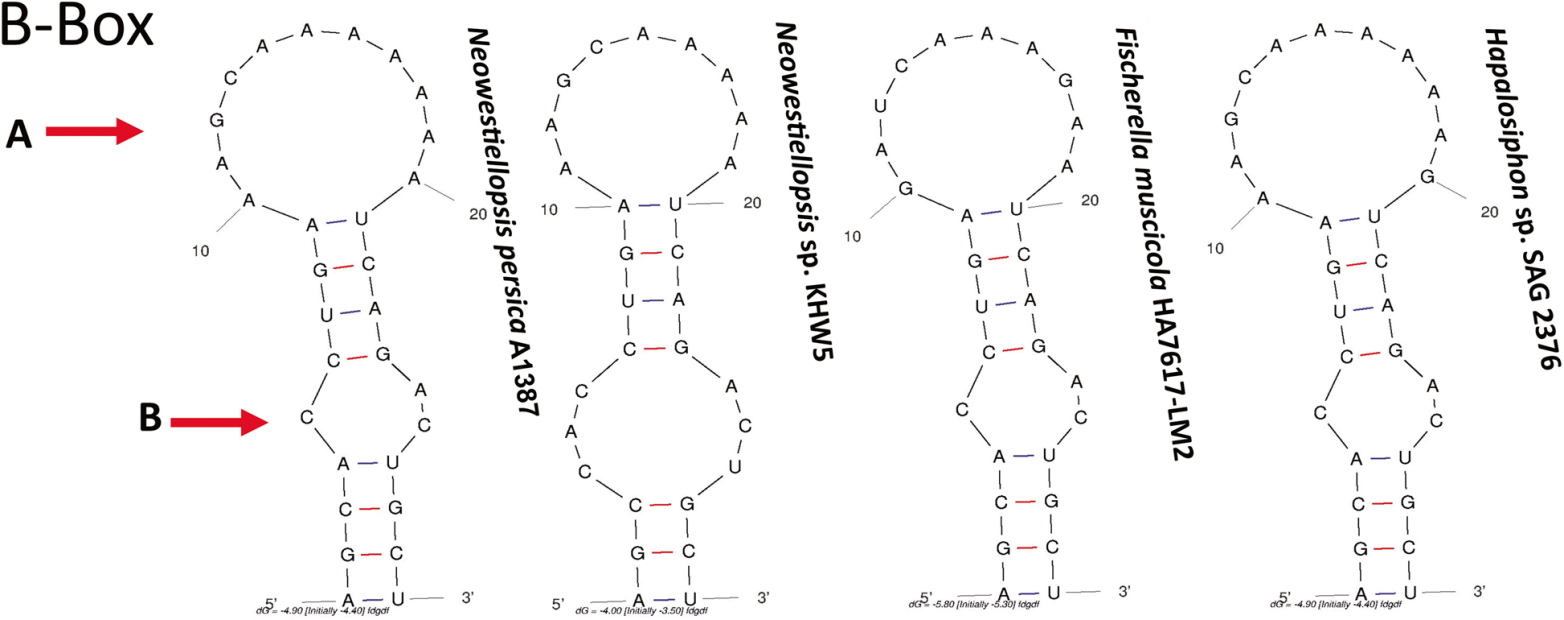
Predicted secondary structures for the Box B helices of studied strain and related taxa. Secondary structures generated from M-fold web server (version 2.3), temperature: 37 °C default; structure: untangled loop fix. Terminal Bilateral Bulge (A), Bilateral Bulge (B)

**Fig. 5:**
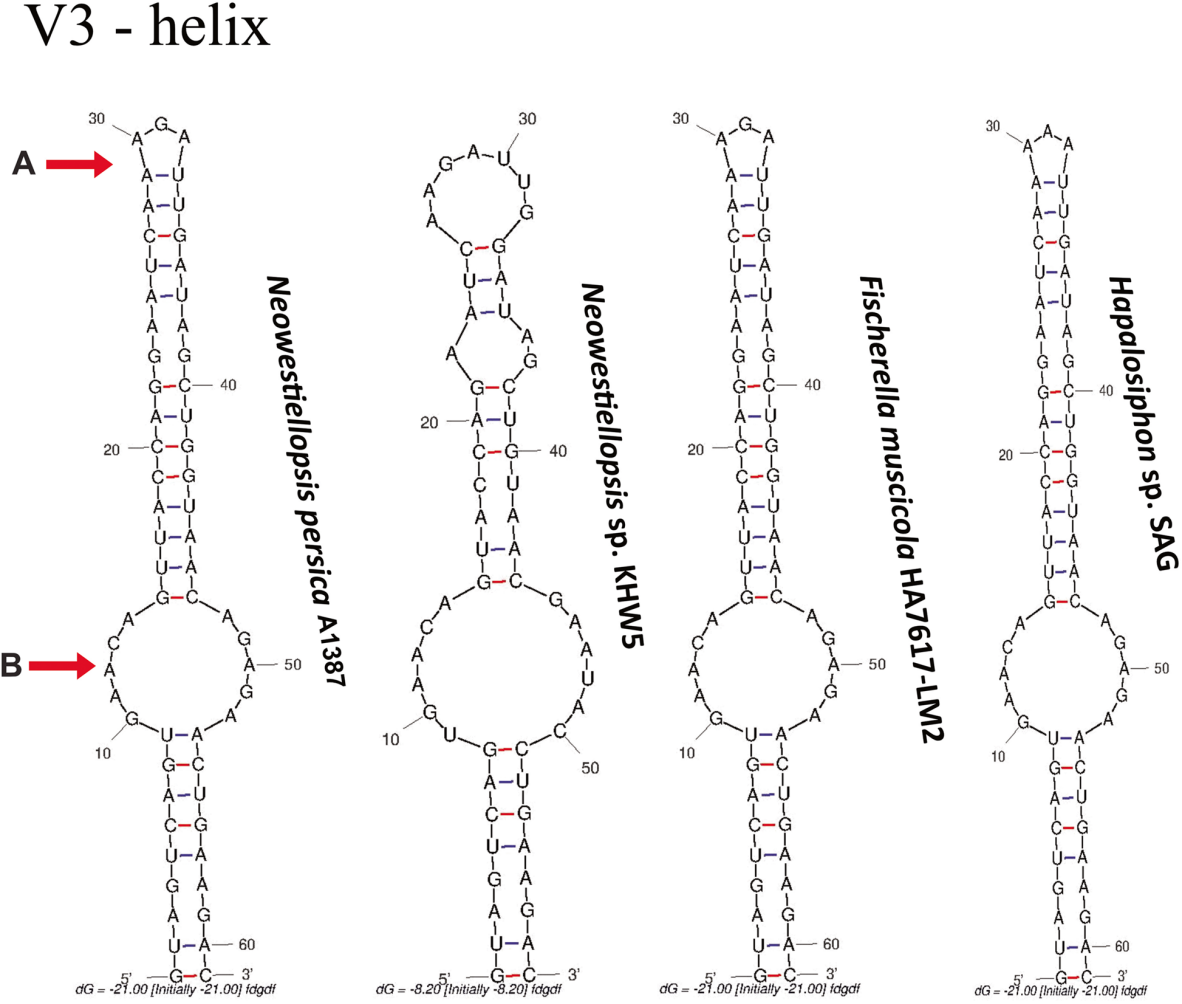
Predicted secondary structures for the V3 region of studied strain and related taxa. Secondary structures generated from M-fold web server (version 2.3), temperature: 37 °C default; structure: untangled loop fix. Terminal Bilateral Bulge (A), Bilateral Bulge (B)

Box-B and V3 helix were nominated by Terminal Bilateral Bulge (A), Bilateral Bulge (B). As to the Box-B, lengths varied from 29 nt (*Neowestiellopsis* sp. KHW5 and *Fischerella muscicola* HA7617-LM2) to 30 nt (*Hapalosiphon* sp. SAG 2376), with studied strain showing a length of 30 nt (Fig. 4). Similarly, the V3 helix was also very different in terms of length and shape between the studied strain and *Neowestiellopsis* sp. KHW5. Lengths varied from 61 nt (*Fischerella muscicola* HA7617-LM2 and *Hapalosiphon* sp. SAG 2376) to 58 nt (*Neowestiellopsis* sp. KHW5), with studied strain showing a length of 61 nt (Fig. 5; Tables S4 and S5).

#### Detection of nostopeptolide biosynthetic genes and cyanotoxin genes

PCR results confirmed potential toxigenicity of the *Neowestiellopsis persica.* Positive amplification was observed for the *mcy* gene of the microcystin biosynthetic gene cluster. Amplification of *nos* gene related to biosynthesis of nostopeptolide, was successfully discovered in studied strain. The length of PCR product for *mcy* G, *mcy* D and *nos* genes, was 700, 1500 and 1500 bp. The partial sequences of the *mcy* G, *mcy* D and *nos* genes were compared with the ones available in the NCBI database (June 2021) using BLASTn. The result of BLASTn showed 87.19% identity to the nostopeptolide biosynthetic gene cluster of *Nostoc* sp. GSV224 (AF204805), 97.96% identity to the polyketide synthase (*mcy*D) gene of *Fischerella* sp. NQAIF311 (KJ364644) and 96.94% identity to the *mcy* G of *Fischerella* sp. CENA161 (KX891213).

#### Nucleotide sequence accession numbers

Sequence data were deposited in the DNA Data Bank of Japan (DDBJ) under the accession numbers MZ327713, MZ345693, MZ345694 and MZ345692 for *16S rRNA, mcy*D, *mcy*G and *nos* (Table S2).

## Discussion

The systematics of the order Nostocales, underwent significant changes in the past years, which brought the description of several new taxa [33–39]. In particular, the *Neowestiellopsis persica* species, has been described for the first time by Kabirnataj et al. [17], originally isolated from Mazandaran (Iran). We isolated the strain of *Neowestiellopsis persica* from the agricultural fields of Kermanshah province. This genus was identified here for the first time, and these fields represent a novel locality of dispersion for this genus. Initially, the genus *Neowestiellopsis* was characterised as nitrogen-fixing heterocytous cyanobacterium, belonging to the order Hapalosiphonaceae, order Nostocales [17]. Nowadays, these genera contain two species *N. bilateralis* and type species *N. persica* [18]. To characterize our strain, we decided to use a polyphasic approach, which comprises the cultivation of cyanobacteria, evaluation of the strain by optical microscopy and the sequencing of 16S rRNA as used in previous work [40]. In addition, we also sequenced the genes potentially responsible for production of toxins: *mycD*, *mycG* and *nos*. We also performed analyses of secondary structures of the 16S rRNA molecule.

Comparative analyses of obtained sequences of 16S rRNA gene region revealed a 100% homology with *Neowestiellopsis persica* SA33 (MF066912.1), however, the phylogenetic analyses based on the same gene region placed our strain distantly apart from *Neowestiellopsis persica*_SA33, within the same supported cluster. This corresponds to the differences in morphological analysis, since the studied strain differs from *Neowestiellopsis_persica*_SA33 in branching, absence of biseriate filaments, presence of monocyte cells, toxicity, and ecology (soil sample and different climate). Unfortunately, the complet ITS regions sequences of studied strain’s closest related taxa, as *Neowestiellopsis_persica*_SA33 is not available and, for this reason, comparisons in this regard could not be conducted. Due to this, the contribution we make to the study of *Neowestiellopsis* strains by reporting the secondary structures of ITS between 16S and 23S rRNA is extremely important. In the original paper, only D1-D1’ helix was compared [17], on the other hand, we compared D1-D1’ helix, D2, D3, BOX B and BOX a region of the 16S-23S ITS. The use of secondary structures of the ITS regions plays a significant role in closely related species identification on the level of population genetic. Within the closely related groups, this region shows extreme variability in size, as well as in sequence [41]. Sequences of the ITS region are used for reconstruction of phylogenetic trees or for comparison of RNA secondary structures among studied strains [42–45]. In the case of multiple operons, the size of the spacer may vary considerably in different species, and even among the different operons within a single cell. The results of these analyses provide additional information on secondary structures of *N. persica* and bring better understanding for future description of new species that could potentially belong to this genus.

The morphological comparison of our strain with type species *N. persica*, shows significant differences. The most common is the type of branching. In our strain, the V type branch was present, which was never described in original type species *N. persica* SA33. In our strains we did not observed the biseriate development of filaments, tapering of terminal cells of branches toward the apex, or the irregular shape of the first cell of branch adjacent to main filament. The average size of cells in our strain is also bigger. The most important difference between our strain and previously described species, is the presence of akinetes and monocytes in our strain. These differences in morphology can be caused by different environmental conditions, from which the strains were isolated.

The presence of microcystin synthetase gene (*mcy*) clusters in our strain suggests that this strain is potentially toxic. This gene cluster was confirmed by the detection and phylogenetic analysis of the *mcyD* and *mcyG* gene sequences. The *mcyD* gene encodes parts of both the b-ketoacyl synthase and the acyltransferase domains, and the *mcyG* encodes for partial C-Methyl transferase domain [46, 47]. Moreover, the entire *mcyD* codes for a polyketide synthase enzyme (McyD) and the entire *mcyE* and *mcyG* genes code for the hybrid enzymes (McyE and McyG) [48]. Two polyketide synthase modules of McyG and McyE with two modular polyketide synthase McyD are directly responsible for the Adda synthesis, the structural component responsible for the toxicity of the hepatotoxin microcystins [49]. In the *mcyD* and *mcyG* phylogenetic tree, the studied species was grouped clearly with members of the Hapalosiphonaceae.

Toxic cyanobacteria that occur in water used for irrigation of fields with edible plants presents a protentional problem for the production of crops and for human health. The use of contaminated water could inhibit the plant growth [50–52]. Through the bioaccumulation in the tissue of irrigated plants, the cyanotoxins can represent a risk for human health [6, 10, 11, 53–55]. Cyanotoxins accumulated in the roots, stems and leaves, which are consumed by herbivorous animals could also pose a health problem.

Based on amplification of *nos* gene in the studied strain, we presume that the studied strain is capable of making nostopeptolides. The gene *Nos* showed homology to ∆1-pyrroline-5-carboxylic acid (P5C) reductases [16]. As numerous studies suggested, these genes have reached parallel assessment in chemical structures of the nostopeptolides [13, 15, 36, 56–58].

The presence of *Neowestiellopsis* in the agricultural areas can lead to the death due to the ingestion of *Crataegus* plant contaminated with cyanobacterial toxins, therefore, it is necessary to studyalso other cyanotoxin genes. Moreover, proactive measures are immediately required to control the organization of cyanobacterial growth and their bioactive secondary compounds in agricultural zones of Iran.

## Conclusion

The polyphasic study of *Neowestiellopsis* A1387 brings new information to *Neowestiellopsis persica* description. Our analyses suggest that our strain has 100% homology to strain *N. persica* SA33, but the phylogenetic analyses based on multi genes, together with analyses of morphology seems to bring evidence that our strain could be a new species. However, this could be not confirmed due to lack of the ITS sequences of the species closely related to our strain. Also, in the case of original strain of *N. persica*, the possibility of toxin production was suggested,, but it was never confirmed. In our study, we investigate the gene clusters *nos*, *mycD* and *mycG*. The presence of *mcy* gene cluster suggests that the strain should be able to produce a hepatotoxin microcystin, and the *nos* gene cluster also suggested the production of nostopeptolides. This information represents a new critical feature for species *N. persica*, which could be used in better identification of new potential species from this gene cluster. Also, the risk evaluation of using the water contaminated with these species, due to this strain toxicity have a significant role in future usage in agriculture.

## Materials and methods

### Sampling, culturing, and phenotypic analysis

Soil samples with different textures (sand, loamy sand, silt loam, silt, clay loam and clay) were chosen from five different areas of the agricultural zones following the pedological map of the Kermanshah province (34° 24′ 32″ N, 47° 00′ 17″). Samples were collected from the surface up to 5 cm deep using a sterilized spatula after removing surface debris. Each sample was aseptically transferred to sterile petri dishes with BG11_0_ liquid media [59].

After 20 days, one or two isolated colonies were selected and washed with sterile deionized water and transferred to 250 ml of fresh liquid BG11_0_ medium. As the culture started to disperse in this medium after 10-12 days, new intact filaments were again plated on the sterile 1.2% agar-solidified BG11_0_ medium by spread plate technique. These steps were repeated until an axenic culture was obtained. Thereafter, the strains were cultured in a 250 ml cotton stoppered Erlenmeyer flask containing 100 ml media, with a pH adjusted to 7.2. Cultures were maintained at 28 ± 2 °C with periodic shaking (twice a day). The culture room was illuminated with ca. 50-55 μmol photons m^− 2^ s^− 1^ with a photoperiod of 14:10 h light: dark cycle [53]. Detailed morphological analyses of these strains were carried out at the time of isolation to avoid difficulties in identification. Subsequently, samples were transferred to 1.2% solidified agar plates with BG11_0_ medium and, once axenic conditions were verified, a morphological observation of the culture utilized an Olympus CX31RTS5 (Olympus, Tokyo, Japan) stereoscope equipped with a QImaging GO-3 digital camera (Teledyne QIMAGING. Surrey, Canada) and Olympus BX43 equipped with manufactured Sc50 digital camera (Olympus, Tokyo, Japan).

The type of filament orientation, sheath (overall distribution and visibility across the trichome), dimensions and shape of the vegetative cells and heterocytes were determined. The heterocytous cyanobacteria strains, which were the most frequent ones, were selected to investigate the differences at morphological and genotypic level, through a polyphasic approach and selected as type species. Fresh culture under accessory number A1387a is deposited in Cyanobacteria Culture Collection (CCC) and exsiccates with accessory numbers A1387b are deposited in herbarium ALBORZ at the Science and Research Branch, Islamic Azad University, Tehran respectively with the accession number A1387.

### Molecular and sequence analysis

Genomic DNA was isolated from 16 days old culture in log phase using the Himedia Ultrasensitive Spin Purification Kit (MB505) following the instructions of the manufacturer, except for the increase of incubation time for the lysis solutions Al and C1, which were set to 60 and 20 min, respectively. DNA fragments within the following genes were amplified using the oligonucleotide primers and PCR programs listed in Table S1. PCR products were checked by electrophoresis on 1% agarose gels (SeaPlaque® GTG®, Cambrex Corporation), using standard protocols. The products were purified directly using the Geneclean® Turbo kit (Qbiogene, MP Biomedicals) and sequenced using the BigDye® Terminator v3.1 cycle sequencing kit (Applied Biosystems, Life Technologies). The partial sequences were compared with the ones available in the NCBI database using BLASTN. The BLAST X tool (blast.ncbi.nlm.nih.gov/Blast.cgi) was used for determination of the *nos*, *mcy G* and *mcy D* genes similarity. The sequences were annotated for the coding regions by the NCBI ORF Finder at NCBI (http://www.ncbi.nlm.nih.gov/gorf/gorf.html) and the ExPASY proteomics server (http://www.expasy.org/tools/dna.html).

### Phylogenetic analysis

The gene sequences obtained in this study, as well as the best hit sequences (> 94% identity) retrieved from GenBank, were first aligned using MAFFT version 7 [60] and then maximum likelihood phylogenetic trees were inferred in IQ-Tree (multicore v1.5.5) [61]. The 133, 20, 43 and 16 sequences were compared in phylogenetic analysis for 16S rRNA, *nos*, *mcy G* and *mcy D* genes, respectively. Optimum models were used as suggested (BIC criterion) after employing model test implemented in IQ-tree (Table S2). Tree robustness was estimated with bootstrap percentages using 100 standard bootstrap and 10,000 ultrafast bootstrap to evaluate branch supports [62].

### 16S-23S rRNA ITS region secondary structure analysis

The sequences corresponding to the D1-D1’ helix, D2, D3, BOX B and BOX A regions of the 16S-23S Internal Transcribe Spacer (ITS) of studied strain were characterized according to the Johansen et al. [32] and tRNA^Ile^ and tRNA^Ala^ were determined according to the tRNAscan-SE 2.0. Comparison of the ITS secondary structures of studied strain and the reference strains were generated using the M-fold web server (version 2.3) [63] under ideal conditions of untangled loop fix and the temperature set to default (37 °C).

## Supplementary Information

**Additional file 1: Supplementary Fig. S1.** Life cycle of the new *Neowestiellopsis* ca. *persica* isolated from Iran. a) Hormogonium, b) filament from hormogonium, c) Akinets, d) Akinets germination, e) multiseriate filament from akinets, f) Monocyte formation from the ends of filaments, g) Monocyte with heterocyte, h) little heteropolar filaments from Monocites, i) heteropolar filaments, j) typical *Neowestiellopsis* filament. **Supplementary Fig. S2.** Phylogenetic position of studied strain (Highlighted in red) and related cyanobacteria based on *mcy G* gene with *Microcystis aeruginosa* (AB110133) as out-group. Numbers near nodes indicate standard bootstrap support (%)/ultrafast bootstrap support (%) for ML analyses. **Supplementary Fig. S3.** Phylogenetic position of studied strain (Highlighted in red) and related cyanobacteria based on *mcy D* gene with *PCC*_7806SL (CP020771) as out-group. Numbers near nodes indicate standard bootstrap support (%)/ultrafast bootstrap support (%) for ML analyses. **Supplementary Fig. S4.** Phylogenetic position of studied strain (Highlighted in red) and related cyanobacteria based on *nos* gene. Numbers near nodes indicate standard bootstrap support (%)/ultrafast bootstrap support (%) for ML analyses. **Table S1.** Target genes and oligonucleotide primers used in this study. **Table S2.** Accession numbers of Sequence data deposited in the DNA Data Bank of Japan. **Table S3.** Comparison of the nucleotides length of the ITS regions of *Neowestiellopsis persica* with reference strains. **Table S4.** Comparison of secondary structure of 16S-23S rRNA (D1-D1^,^ helix and Box-B helix) between the *Neowestiellopsis persica* and related taxa. **Table S5.** Comparison of secondary structure of 16S-23S rRNA (D2^,^ helix and V3) between the Neowestiellopsis persica and related taxa.

## Data Availability

The strain culture is deposit in Cyanobacteria Culture Collection (CCC) under the accessory number A1387a. Strain exsiccated are deposited in herbarium ALBORZ at the Science and Research Branch, Islamic Azad University, Teheran under accessory number A1387b Genetic data are available in the DNA Data Bank of Japan repository under accessory numbers MZ327713 for 16S rRNA (https://www.ncbi.nlm.nih.gov/nuccore/MZ327713), MZ345693 for *mcyD* gene (https://www.ncbi.nlm.nih.gov/nuccore/MZ345693), MZ345694 for mcyG gene (https://www.ncbi.nlm.nih.gov/nuccore/MZ345694) and MZ345692 for nos gene (https://www.ncbi.nlm.nih.gov/nuccore/MZ345692).

## Abbreviations

S: Seconds
min: Minutes
h: Hour
ITS: Internal transcribed spacer
16S rRNA gene: Small subunit ribosomal gene
*nos* gene: Gene cluster coding for the biosynthesis of Nostopeptolides
*mcy*D and mcyG: Microcystin biosynthesis (*mcy*) gene clusters
