# Supplementary material for: A new strain of Neowestiellopsis (Hapalosiphonaceae): first observation of toxic soil cyanobacteria from agricultural fields in Iran
Source: BMC Microbiol. 2022 Apr 18;22:107. doi: 10.1186/s12866-022-02525-x (PMC9014592; doi:10.1186/s12866-022-02525-x)
Supplement: Supplementary file 1 — Additional file 1: Supplementary Fig. S1. Life cycle of the new Neowestiellopsis ca. persica isolated from Iran. a) Hormogonium, b) filament from hormogonium, c) Akinets, d) Akinets germination, e) multiseriate filament from akinets, f) Monocyte formation from the ends of filaments, g) Monocyte with heterocyte, h) little heteropolar filaments from Monocites, i) heteropolar filaments, j) typical Neowestiellopsis filament. Supplementary Fig. S2. Phylogenetic position of studied strain (Highlighted in red) and related cyanobacteria based on mcy G gene with Microcystis aeruginosa (AB110133) as out-group. Numbers near nodes indicate standard bootstrap support (%)/ultrafast bootstrap support (%) for ML analyses. Supplementary Fig. S3. Phylogenetic position of studied strain (Highlighted in red) and related cyanobacteria based on mcy D gene with PCC_7806SL (CP020771) as out-group. Numbers near nodes indicate standard bootstrap support (%)/ultrafast bootstrap support (%) for ML analyses. Supplementary Fig. S4. Phylogenetic position of studied strain (Highlighted in red) and related cyanobacteria based on nos gene. Numbers near nodes indicate standard bootstrap support (%)/ultrafast bootstrap support (%) for ML analyses. Table S1. Target genes and oligonucleotide primers used in this study. Table S2. Accession numbers of Sequence data deposited in the DNA Data Bank of Japan. Table S3. Comparison of the nucleotides length of the ITS regions of Neowestiellopsis persica with reference strains. Table S4. Comparison of secondary structure of 16S-23S rRNA (D1-D1, helix and Box-B helix) between the Neowestiellopsis persica and related taxa. Table S5. Comparison of secondary structure of 16S-23S rRNA (D2, helix and V3) between the Neowestiellopsis persica and related taxa. [file 12866_2022_2525_MOESM1_ESM.zip › Supplementary file.docx]

**Sub figure legend:**

**Supplementary Fig. S1** Life cycle of the new *Neowestiellopsis* ca. *persica* isolated from Iran

a) Hormogonium, b) filament from hormogonium, c) Akinets, d) Akinets germination, e) multiseriate filament from akinets, f) Monocyte formation from the ends of filaments, g) Monocyte with heterocyte, h) little heteropolar filaments from Monocites, i) heteropolar filaments, j) typical *Neowestiellopsis* filament.

**Supplementary Fig. S2** Phylogenetic position of studied strain (Highlighted in red) and related cyanobacteria based on *mcy G* gene with *Microcystis aeruginosa* (AB110133) as out-group. Numbers near nodes indicate standard bootstrap support (%)/ultrafast bootstrap support (%) for ML analyses.

**Supplementary Fig. S3** Phylogenetic position of studied strain (Highlighted in red) and related cyanobacteria based on *mcy D* gene with *PCC*_7806SL (CP020771) as out-group. Numbers near nodes indicate standard bootstrap support (%)/ultrafast bootstrap support (%) for ML analyses.

**Supplementary Fig. S4** Phylogenetic position of studied strain (Highlighted in red) and related cyanobacteria based on *nos* gene. Numbers near nodes indicate standard bootstrap support (%)/ultrafast bootstrap support (%) for ML analyses.

**Table S1.** Target genes and oligonucleotide primers used in this study.

| **Primer name** | **Target gene** | **Sequence 5´ - 3´** | **Thermal profile** | **Reference** | |
| --- | --- | --- | --- | --- | --- |
| **pA**  **B23S** | 16S rRNA  16S rRNA | AGAGTTTGATCCTGGCTCAG  CTTCGCCTCTGTGTGCCTAGGT | 94˚C, 3 min 30 x (94˚C, 30 s; 55˚C, 40 s; 72˚C, 1.30 min) 72˚C, 3 min 4˚C, ∞ | Taton *et al*. 2003  Taton *et al*. 2003 | |
| **ITS-F**  **ITS-R** | 16S-23S rRNA ITS  16S-23S rRNA ITS | TGTACACACCGCCCGTC  CTCTGTGTGCCTAGGTATCC |  | Iteman *et al*. 2000  Iteman *et al*. 2000 | |
| **nosF**  **nosR** | NOS  NOS | GAAACCTGTTACAACTGCTGGTATTG  TGAACBCCAGCATCAATCAT | 94˚C, 5 min 35 x (94˚C, 60 s; 56˚C, 60 s; 72˚C, 60 s) 72˚C, 10 min 4˚C, ∞ | Luesch *et al.* (2003)  Luesch *et al.* (2003) |  |
| **mcyGF**  **mcyGR** | *mcy*G  *mcy*G | GAAATTGGTGCGGGAACTGGAGT  TTGAGCAACAATGATACTTTGCTG | 95˚C, 5 min 34 x (95˚C, 30 s; 53˚C, 30 s; 72˚C, 60 s) 72˚C, 5 min 4˚C, ∞ | Fewer *et al.* 2007  Fewer *et al.* 2007 | |
| **mcyDF**  **mcyDR** | *mcy*D  *mcy*D | GCTCAAGAAAAATTACATCAAG  TTAAAGGAGAATGAAAAGCATGAGA |  | Rantala *et al.* 2004  Rantala *et al.* 2004 | |

**Table S2.** Accession numbers of Sequence data deposited in the DNA Data Bank of Japan.

| **Target gene** | **Strain** | **Nucleotide ID** | **Number of nucleotides** | **Number of amino acids** | **Tree model** |
| --- | --- | --- | --- | --- | --- |
| ***16S rRNA*** | A1387 | MZ327713 | 2073 | - | TVM+F+I+G4 |
| ***mcy*D** | A1387 | MZ345692 | 972 | 324 | TVMe+I+G4+F |
| ***mcy*G** | A1387 | MZ345693 | 524 | 174 | TIM2+F+I+G4 |
| ***nos*** | A1387 | MZ345694 | 342 | 114 | TMP3U+G4+F |

**Table S3:** Comparison of the nucleotides length of the ITS regions of *Neowestiellopsis persica* with reference strains.

| **V3** | **D4+spacer** | **D 4** | **BOX A** | **Post BOX B spacer** | **BOX B** | **Pere BOX B spacer** | **TrRNA^Ala^ gene** | **spacer+V2+spacer** | **trRNA^Ile^ gene** | **D3 with spacer** | **spacer+D2+spacer** | **D1-D1^,^ helix** | ***Studied strain and reference strains*** |
| --- | --- | --- | --- | --- | --- | --- | --- | --- | --- | --- | --- | --- | --- |
| 61 | 22 | 8 | 11 | 17 | 30 | 28 | 73 | 74 | 74 | 5 | 38 | 71 | (MZ327713) *Neowestiellopsis persica* A1387 |
| - | - | - | - | - | - | - | - | - | - | - | - | 71 | (MF066911) *Neowestiellopsis bilateralis* SA16 |
| 58 | 27 | 6 | 10 | 16 | 29 | 31 | 73 | 74 | 73 | 5 | 38 | 71 | (MN656995) *Neowestiellopsis* sp. KHW5 |
| 61 | 22 | 9 | 11 | 17 | 29 | 28 | 73 | 82 | 74 | 5 | 38 | 71 | (KF417427) *Fischerella muscicola* HA7617-LM2 |
| 61 | 22 | 9 | 11 | 17 | 30 | 28 | 73 | 89 | 74 | 5 | 38 | 71 | (MK953008) *Hapalosiphon* sp. SAG 2376 |
| - | - | - | - | - | - | - | - | - | - | - | - | 61 | (MF066912) *Neowestiellopsis* persica SA33 |
| - | - | - | - | - | - | - | - | - | - | - | - | 66 | (KY883375) *Westiellopsis ramosa* HPS |

**Table S4:** Comparison of secondary structure of 16S-23S rRNA (D1-D1^,^ helix and Box-B helix) between the *Neowestiellopsis persica* and related taxa

| ***Studied strain and reference strains*** | ***D1-D1^,^ helix*** | | | | | ***BOX B*** | | |  |  |
| --- | --- | --- | --- | --- | --- | --- | --- | --- | --- | --- |
|  | **Terminal Bilateral Bulge (A)** | **Bilateral Bulge (B)** | **Unilateral Bulge (C)** | **Basal Clamp (D)** | **Terminal Bilateral Bulge (A)** | | **Bilateral Bulge (B)** | | |  |
|  | **Number of nucleotides** | **Number of loops** | **Number of loops** | **Number of nucleotides** | | **Number of nucleotides** | | **Number of nucleotides** | | |
| (MZ327713) *Neowestiellopsis persica* A1387 | 8 | 1 | 2 | 12 | | 13 | | 7 | | |
| (MF066911) *Neowestiellopsis bilateralis* SA16 | 8 | 1 | 2 | 12 | | - | | - | | |
| (MN656995) *Neowestiellopsis* sp. KHW5 | 8 | 1 | 2 | 12 | | 11 | | 10 | | |
| (KF417427) *Fischerella muscicola* HA7617-LM2 | 8 | 1 | 2 | 12 | | 12 | | 7 | | |
| (MK953008) *Hapalosiphon* sp. SAG 2376 | 8 | 1 | 2 | 12 | | 13 | | 7 | | |
| (MF066912) *Neowestiellopsis persica* SA33 | 8 | 1 | 2 | 12 | | - | | - | | |
| (KY883375) *Westiellopsis ramosa* HPS | 10 | 2 | 1 | 12 | | - | | - | | |

**Table S5:** Comparison of secondary structure of 16S-23S rRNA (D2^,^ helix and V3) between the Neowestiellopsis persica and related taxa

| ***Studied Strain and reference strains*** | **D2^,^ helix** | | | **V3** | | | |  |
| --- | --- | --- | --- | --- | --- | --- | --- | --- |
|  | **Terminal Bilateral Bulge (A)** | | **Basal Clamp (D)** | **Terminal Bilateral Bulge (A)** | | | **Bilateral Bulge (B)** |  |
|  | **Number of nucleotides** | | **Number of nucleotides** | **Number of nucleotides** | | | **Number of nucleotides** |  |
| (MZ327713) *Neowestiellopsis persica* A1387 | 8 | 20 | | | 5 | 14 | | |
| (MN656995) *Neowestiellopsis* sp. KHW5 | 8 | 20 | | | 9 | 16 | | |
| (KF417427) *Fischerella muscicola* HA7617-LM2 | 8 | 20 | | | 5 | 14 | | |
| (MK953008) *Hapalosiphon sp*. SAG 2376 | 8 | 20 | | | 5 | 14 | | |
| (MF066912) *Neowestiellopsis persica* SA33 | - | - | | | - | - | | |
| (KY883375) *Westiellopsis ramosa* HPS | - | - | | | - | - | | |
